# Palmitate but Not Oleate Exerts a Negative Effect on Oxygen Utilization in Myoblasts of Patients with the m.3243A>G Mutation: A Pilot Study

**DOI:** 10.3390/life10090204

**Published:** 2020-09-16

**Authors:** Leila Motlagh Scholle, Helena Schieffers, Samiya Al-Robaiy, Annemarie Thaele, Diana Lehmann Urban, Stephan Zierz

**Affiliations:** 1 Department of Neurology, Martin-Luther-University Halle-Wittenberg, 06120 Halle (Saale), Germany; helena@schieffers.de (H.S.); annemarie.thaele@uk-halle.de (A.T.); stephan.zierz@medizin.uni-halle.de (S.Z.); 2 Center for Basic Medical Research, Martin-Luther-University Halle-Wittenberg, 06120 Halle (Saale), Germany; samiya.al-robaiy@uk-halle.de; 3 Department of Neurology, Ulm University, 89070 Ulm, Germany; diana.lehmann@rku.de

**Keywords:** fatty acid oxidation, palmitate, oleate, m.3243A>G mutation

## Abstract

It is known that exposure to excess saturated fatty acids, especially palmitate, can trigger cellular stress responses interpreted as lipotoxicity. The effect of excessive free fatty acids on oxidative phosphorylation capacity in myoblasts of patients with the m.3243A>G mutation was evaluated with the mitochondrial (Mito) stress test using a Seahorse XF96 analyzer. ß-oxidation, measured with the Seahorse XF96 analyzer, was similar in patients and controls, and reduced in both patients and controls at 40 °C compared to 37 °C. Mito stress test in the absence of fatty acids showed lower values in patients compared to controls. The mitochondrial activity and ATP production rates were significantly reduced in presence of palmitate, but not of oleate in patients, showing a negative effect of excessive palmitate on mitochondrial function in patients. Diabetes mellitus is a frequent symptom in patients with m.3243A>G mutation. It can be speculated that the negative effect of palmitate on mitochondrial function might be related to diacylglycerols (DAG) and ceramides (CER) mediated insulin resistance. This might contribute to the elevated risk for diabetes mellitus in m.3243A>G patients.

## 1. Introduction

Fatty acid oxidation (FAO) provides the major source of energy supply for skeletal muscles in situations requiring simultaneous glucose sparing, and major energy supply, such as prolonged fasting or exercise. ß-oxidation is the main pathway of fatty acid catabolism in mitochondria [1]. The accumulation of oversupplied fatty acids (FAs) in different tissues, such as the pancreas, liver, and skeletal muscle, might lead to a cellular dysfunction in these tissues, and an apoptotic cell death, commonly referred to as “lipotoxicity” [2,3]. The toxic effects of FAs seem to be dependent on their chain length and degree of saturation. The two-common long-chain saturated FAs (SFA), palmitate (C16:0) and stearate (C18:0), are known to be the most lipotoxic ones. In contrast, monounsaturated FAs such as oleate (C18:1), have been reported to protect against the above-mentioned SFA-induced toxicity [2,4]. Moreover, saturated FAs have worsened the insulin-resistance, while monounsaturated and polyunsaturated ones improved it [5].

The effect of temperature on the functional properties of skeletal rat muscle mitochondria was reported in one study [6]. Increasing the assay temperature in above-mentioned study within the range of 25–42 °C increased the mitochondrial respiratory chain activities, resulting in an elevated phosphorylation rate. They reported a temperature-induced decrease in oxidative phosphorylation (OXPHOS) efficiency as well.

It has been shown that the increase of temperature from 35 °C to 40 °C induced the uncoupling of substrate oxidation from adenosine diphosphate (ADP) phosphorylation, and decreased the efficiency of mitochondria to produce adenosine triphosphate (ATP) in skeletal myofibers. This uncoupling effect was more pronounced for FAs than for carbohydrates as substrate [7]. It is already known that a reduction in substrate oxidation, caused by mitochondrial dysfunctions, leads to a lipid accumulation, such as the deposition of lipid mediators, such as diacylglycerols (DAG) and ceramides (CER). Both, DAG and CER are reported to inhibit insulin signaling [8,9,10].

The m.3243A>G point mutation in the *MT-TL1* gene (encoding mt-tRNA^Leu(UUR)^) can be found in approximately 80% of patients with mitochondrial encephalopathy, lactic acidosis, and stroke like episodes (MELAS)-syndrome. In these patients, the ATP production rate was significantly less than in controls [11] and the mitochondrial respiration is known to be impaired [12]. Diabetes mellitus frequently accompanies the m.3243A>G mutation [13]. Thus, the evaluation of effects of saturated free fatty acids (FFAs), which could lead to an insulin resistance, might be relevant for these patients.

The aim of the present study was to evaluate the oxidative phosphorylation and ß-oxidation pathway in patients harboring the m.3243A>G mutation. It was of interest to examine whether palmitate and oleate show a toxic or protective effect on OXPHOS in myoblasts of patients with the m.3243A>G mutation. Moreover, the effect of a fever-stimulating temperature on ß-oxidation was simulated in 40 °C cultured cells in both, m.3243A>G patients and controls.

## 2. Materials and Methods

### 2.1. Human Myoblasts

Muscle primary cells from five patients harboring the genetically confirmed m.3243A>G mutation and 5 controls were provided by the Muscle Tissue Culture Collection (MTCC) at the Friedrich-Baur-Institute (Department of Neurology, Ludwig Maximilian University, Munich, Germany; part of the German network on muscular dystrophies, MD-NET, partner of EuroBioBank) [14].

Cells were collected and processed by MTCC in compliance with all applicable laws, rules, regulations, and other requirements of any applicable governmental authority. The cells were grown in skeletal muscle cell growth medium (PromoCell, Heidelberg, Germany) supplemented with 10% fetal bovine serum (FBS). Further details are given in Table 1 and as described earlier [12]. As controls served 5 patients (2 males, 3 females), who had muscle biopsy for diagnosis of a suspected neuromuscular disorder. They were deemed ‘normal controls’ if they were ultimately found to have no muscle disease by combined clinical and histological criteria. Age of controls ranged from 35 to 53 years.

### 2.2. Metabolic Function Measurements with the Seahorse XF96 Cell Analyzer

#### 2.2.1. Mito Stress Test

To evaluate mitochondrial function, the Mito Stress test was performed using a Seahorse XF96 Cell Analyzer according to the manufacturer’s recommendations. Briefly, myoblasts from patients and controls were seeded to Seahorse XF96 cell culture microplates (2.5 × 10^4^ cells per well) in skeletal muscle cell growth medium supplemented with 10% fetal bovine serum (FBS). After a 24-h incubation at 37 °C, the cells were washed twice with a pre-warmed assay medium (XF base medium supplemented with 10 mM glucose, 2 mM glutamine, and 1 mM sodium pyruvate; pH 7.4).

Oxygen-consumption rate (OCR) values were simultaneously measured following sequential injections of inhibitors of mitochondrial oxidative phosphorylation: (I) oligomycin (2 μM), an ATP synthase inhibitor; (II) carbonyl cyanide p-(trifluoromethoxy) phenylhydrazone (FCCP, 2 μM), an uncoupler; (III) antimycin A (0.5 μM) (inhibitor of complex III) and (IV) rotenone (0.5 μM) (inhibitor of complex I), inhibiting uncoupled respiration. Key parameters of mitochondrial function such as basal respiration (BR), ATP production rate (ATP-R) and spare respiratory capacity (SRC) were analyzed using the above-described measurements. The data was normalized to cell numbers by measurement of Hoechst dye staining of nuclei with excitation and emission wavelengths 355 nm and 465 nm using a Tecan Infinite TM M1000 and plotted as OCR (pmol/min/U fluorescence/well ± SD), accordingly.

##### The Effect of FFAs on the Mitochondrial Respiration

To test the effect of FFAs on the mitochondrial respiration, patient and control myoblasts were plated (as described in Section 2.2.1) 16 h prior to Mito stress test measurements, Bovine serum albumin (BSA)-conjugated palmitic acid or oleic acid (ratio FFA: BSA = 6:1) was added to the media. The final concentration of oleate or palmitate was 300 μM. Comparable amounts of BSA were added in the untreated group.

#### 2.2.2. Fatty Acid Oxidation

To assess the ability of myoblasts to oxidize exogenous fatty acids, the oxygen-consumption rate (OCR) was analyzed using a XF96 Cell Analyzer according to the manufacturer’s protocol (Seahorse Bioscience). For this purpose, the myoblast of patients and controls were plated (as described in Section 2.1) 16-h prior to measurements, growth medium was replaced by substrate-limited medium (Dulbecco’s modified Eagle’s medium (DMEM) with 0.5 mM glucose, 1.0 mM glutamine, 0.5 mM carnitine, and 1% FBS) and incubated at 37 °C or 40 °C. Moreover, 45 min before the beginning of OCR measurement, the medium was changed into pre-warmed FAO Assay Medium (111 mM NaCl, 4.7 mM KCl, 2.0 mM MgSO_4_, 1.2 mM Na_2_HPO_4_, 2.5 mM glucose, 0.5 mM carnitine, and 5 mM HEPES). Just before measurements, palmitic acid or oleic acid were added to a final concentration of 167 μM total FA (bound in a 6:1 molar ratio to BSA) as substrate. The data were normalized to cell numbers.

All Seahorse experiments were repeated at least twice. All data shown are the means ± SD of at least 3 different wells per group.

All chemicals used for these assays were obtained from Sigma Aldrich (St Louis, MO, USA).

### 2.3. Statistical Analysis

Statistical analysis, calculation, and visualization were performed using Prism 8 (GraphPad, San Diego, CA, USA). Analysis of correlation was carried out using a two-way analysis of variance (ANOVA) followed by Tukey’s post hoc test. Significance was set at *p* = 0.05. The statistical tests chosen were predetermined by the size of the study group and the numerical range of values.

### 2.4. Ethical Statement

The study was conducted in accordance with the Declaration of Helsinki and was approved by the local Ethical Committee of the Medical Faculty of Martin Luther University of Halle-Wittenberg (Project identification codes 215/20.01.10/3 and 2020-019).

## 3. Results

### 3.1. Metabolic Function Measurements with the Seahorse XF96 Cell Analyzer

#### 3.1.1. Mito Stress Test

**Figure 1:**
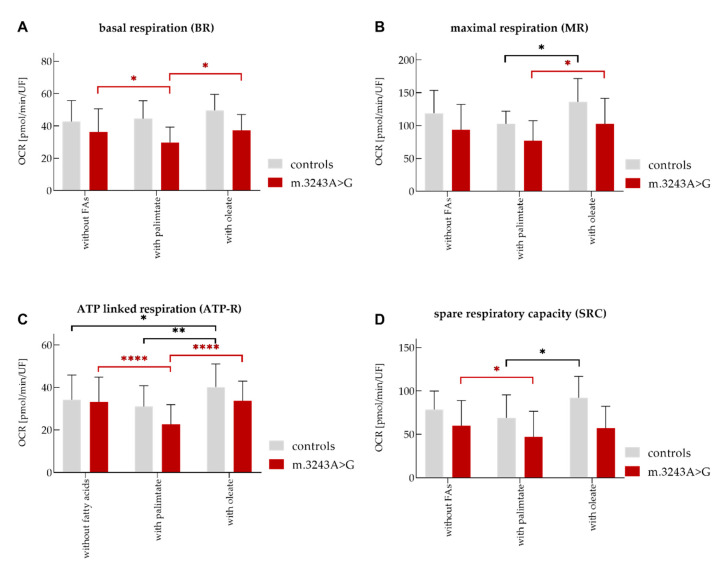
Evaluation of mitochondrial function using a Seahorse XF96 Cell Analyzer in myoblasts from patients (*n* = 5) and controls (*n* = 5). Key parameters of mitochondrial function were analyzed as described in methods. (**A**) basal respiration (BR), (**B**) maximal respiration (MR), (**C**) ATP linked respiration (ATP-R), and (**D**) spare respiratory capacity (SRC). In some experiments, FAs oleate or palmitate were added as excessive nutrients. The significant differences between patients and controls are presented in Table 2. The significance between values without FAs or after treatment with them is only shown if there are significant differences: * = *p*-values ≤ 0.05, ** = *p*-values ≤ 0.01, **** = *p*-values ≤ 0.0001. OCR: oxygen consumption rate, FAs: Fatty acids, UF: unit fluorescence/well.

Without any additional FAs, almost all important parameters of mitochondrial function, such as basal respiration (BR), maximal respiration (MR) and spare respiratory capacity (SRC) were significantly lower in patients compared to controls. Only the ATP linked respiration rate (ATP-R) was not significantly different in patients and controls (Figure 1, Table 2).

#### 3.1.2. The Effect of FFAs on Mitochondrial Respiration

##### Differences between Patients and the Controls

After treatment of myoblasts with palmitate or oleate, the key respiratory parameters BR and SRC were still significantly higher in controls compared to patients. MR was significantly higher in controls than in patients only upon addition of oleate (Table 2).

##### The Effect of Palmitate

The treatment of patient’s myoblasts with palmitate for 16 h prior to measurement led to a significant reduction of BR, SRC, and ATP-R values compared to the values without FAs. In contrast, controls showed similar BR, MR, SRC, and ATP-R values to those without FAs after treatment with palmitate (Figure 1, Table 3).

##### The Effect of Oleate

The treatment of cells with oleate only led to a significant increase of ATP-R in controls compared to the values without FAs. However, BR, MR, and SRC remained unchanged (Figure 1, Table 3).

BR was significantly higher in patients in presence of oleate compared to those in presence of palmitate. MR and ATP-R were higher in both patients and controls after treatment of the cells with oleate, compared to values in presence of palmitate (Table 3).

#### 3.1.3. Fatty Acid Oxidation (FAO)

##### The Difference between Patients and the Controls

**Figure 2:**
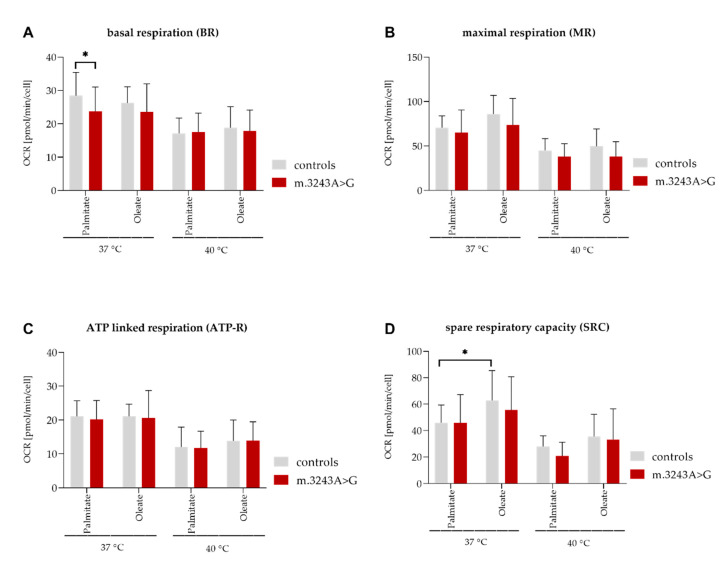
Evaluation of ß-oxidation using a Seahorse XF96 Cell Analyzer in myoblasts from patients (*n* = 5) and controls (*n* = 5). The measurements were performed at 37 °C or 40 °C as described in methods. (**A**) basal respiration (BR), (**B**) maximal respiration (MR), (**C**) ATP linked respiration (ATP-R), and (**D**) spare respiratory capacity (SRC) by use of palmitate or oleate as substrate. BR, MR, ATP-R, and SRC (key parameters) were significantly reduced upon increased temperature from 37 °C to 40 °C in both patients and controls (significance shown in Table 5) * = *p*-values ≤ 0.05. OCR: oxygen consumption rate, UF: unit fluorescence/well.

Independent of the applied substrate (oleate or palmitate), MR, ATP-R, and SRC were similar in patients and controls at 37 °C and 40 °C. BR was higher in controls than in patients, however, only at 37 °C and in presence of palmitate (Figure 2, Table 4). The increase of temperature from 37 °C to 40 °C led to a significant decrease in all key parameters (BR, MR, ATP-R, and SRC) in both, patients and controls (Table 5).

##### The Difference between Palmitate and Oleate

The important parameters BR, MR, ATP-R, and SRC were mostly similar with palmitate or oleate as substrate. Only SRC was higher in controls in the presence of oleate as substrate compared to the value resulted from palmitate as substrate at 37 °C (Table 6).

## 4. Discussion

Mutations of the mitochondrial DNA (mtDNA) involve different organs. These genetic changes can affect the translation of mtDNA-encoded proteins, including respiratory chain complexes [15,16]. The m.3243A>G mutation has been described to be of strong diabetogenic nature [17]. Elevated FFAs are discussed to be a major cause of insulin resistance in skeletal muscle and liver [18].

It is generally accepted, that saturated FAs like palmitate induce insulin resistance, whereas the monounsaturated ones like oleate increase insulin sensitivity in diabetic patients and healthy individuals [4]. It has been shown, that oleate, but not palmitate, increased the expression of genes related to the FAO pathway in a sirtuin (Sirt)1-peroxisome proliferator-activated receptor gamma coactivator (PGC)-1α dependent manner. This, in turn, led to an increase in complete FA oxidation in mice skeletal muscle [19]. There are other reports about the preventive effect of oleate on saturated-fatty-acid-induced endoplasmic reticulum (ER) stress, inflammation, and insulin resistance through adenosine monophosphate-activated protein kinase (AMPK). In the above-mentioned study, oleate, in contrast to palmitate, did not increase the levels of ER stress markers, which is involved in the link between lipid-induced inflammation and insulin resistance [20].

In the present study, taking palmitate as standard substrate for FAO measurements and oleate as a comparison, the data showed generally similar ß-oxidation rates in m.3243A>G patients and controls. Both, patients and controls showed decreased parameters in this pathway at 40 °C compared to 37 °C. ATP-R was reduced to about 35–45% at 40 °C compared to that at 37 °C for both, patients and controls.

Using glucose as substrate for the Mito stress test, all key parameters of mitochondrial respiration were significantly lower in patients than in controls.

The long-chain FFAs are poorly soluble in aqueous solutions. Moreover, in vitro exposure to high levels of FFAs might lead to lipotoxicity and cellular dysfunction [21,22]. The FAs used in the present study were conjugated with BSA in all experiments (ratio FFA: BSA = 6:1).

The treatment of fibroblasts from healthy controls with palmitic acid for 16 h prior to OCR measurement has been reported to significantly increase the MR and SRC for about 20% and 45%, accordingly [23]. However, oleic acid displayed no effect on OCR in the above-mentioned study. In the present study, the extra addition of FAs oleate and palmitate affected the OCR key parameters in Mito stress test differently. While oleate generally showed positive effects on OXPHOS values, palmitate had an impaired them. Considering the values obtained in presence of FAs excess, oleate seemed to show a slightly positive effect on respiratory factors in patients. After addition of oleate, ATP-R was about 30% higher in controls compared to the values with palmitate. In contrast, the negative effect of palmitate excess was significant in patients. MR and ATP-R were reduced about 17% and 30% in patients after addition of palmitate, accordingly.

The different effects in myoblasts in presence of oleate and palmitate could be explained as follows: before performance of FAO measurements, the cells are cultured for 16 h in a nutrient restricted medium (Section 2.2.2). Thus, the cells are forced to take the FAs as the only present substrate to survive. On the other hand, by Mito stress test, the cells have sufficient nutrients and the added FAs (oleate and palmitate) count as excessive substrates. FAs are the major energy source for skeletal muscle. However, the balance between energy demand, uptake, and β-oxidation of FAs should be regulated. An imbalance between fatty acid uptake and β-oxidation might lead to an insulin resistance [24]. The elevated lipid levels, which exceed the cell’s capacity to store or utilize FAs, can, as well, lead to a lipotoxic response to activate stress pathways and apoptosis. For ß-oxidation in mitochondria, both, saturated and unsaturated long-chain FAs are used as substrate. However, only long-chain saturated fatty acyl CoAs serve as substrates for de novo ceramide synthesis, which is involved in initiation of apoptosis [25].

A reduction in palmitic acid, but not in oleic acid oxidation, has been reported in myotubes of patients with diabetes type II compared to controls [26]. It has been shown that monounsaturated fatty acids, such as oleic acid, are metabolized and then accumulated in the form of low-toxic triacylglycerol (TAG). However, a large amount of palmitate inhibits the TAG synthesis at the DAG stage, which leads to the accumulation of DAG in the cell [27,28]. Based on these findings, a substitution of palmitate and other saturated or unsaturated FFAs has been recommended in patients with diabetes type II to reduce the accumulation of DAG and TAG to not promote insulin resistance [26].

The data confirmed the reported negative effect of palmitate on respiratory function of cells. This negative effect was more pronounced in m.3243A>G patients, which led to approximately 30% reduction of ATP production. The controls seemed to benefit slightly more than patients from the positive effect of oleate clearing the excess of saturated FAs via increasing the FA Oxidation. This counteracts with inflammation and insulin resistance in skeletal muscle [19]. Nevertheless, this positive effect should be noticed for keeping the balance of the body weight in over nutrition or pathological states, such as mitochondrial disorders.

An induced mitochondrial reactive oxygen species (ROS) production, mitochondrial dysfunction, and insulin resistance in skeletal muscle cells have been reported upon supplementation of palmitate, but not of oleate. Oleate should even have a protective benefit against palmitate-induced insulin resistance and might enhance the mitochondrial function, protecting against apoptosis, and increasing insulin sensitivity [29]. The analysis of potential apoptosis and ROS production might be evaluated in upcoming projects.

## 5. Conclusions

The data in the present study showed different effects of palmitate and oleate on oxygen utilization in myoblasts of both m.3243A>G patients and controls. The myoblasts, indeed, showed similar ß-oxidation values using palmitate or oleate as substrate. However, the presence of excessive palmitate showed a negative effect on respiratory rates of patients. This could confirm the already reported negative effect of excessive palmitate on mitochondrial function in other studies. Since saturated fatty acids increase the insulin resistance, these results might reveal why the patients harboring the m.3243A>G mutation have a higher risk for developing of diabetes type II.

Limitations: the study was performed on a small study population. In particular, in the case of the 70-year-old patient listed in the cohort, the aging might have an effect on mitochondrial dysfunction. However, it has to be noticed that mitochondrial diseases are rare diseases with a huge variety of mutations and the present study should be considered as a pilot one; it is not always responsible to perform a tissue/muscle biopsy. It is known that patients harboring the m.3243A>G mutation have a higher risk of developing diabetes mellitus. However, in the present study, only one patient is diagnosed with diabetes mellitus (Patient P1).

## Figures and Tables

**Table 1:** Sex and age of five patients with genetically confirmed m.3243A>G mutation and five healthy controls as previously described [12], F: female, M: male.

	Gender	Age at Biopsy
**Patients**		
P 1	M	43
P 2	M	42
P 3	F	70
P 4	M	34
P 5	F	40
**Controls**		
C 1	F	50
C 2	M	53
C 3	F	40
C 4	M	35
C 5	F	49

**Table 2:** Comparison of the mean values of key parameters for mitochondrial function (basal, MR, ATP production, and SRC) in myoblasts of patients (*n* = 5) and controls (*n* = 5) with or without 300 µM palmitate or oleate. Significant differences between patients and controls are shown if *p* ≤ 0.05.

	Mito Stress Test								
				+Palmitate			+Oleate		
	Controls (Mean)	Patients (Mean)	*p*-Value	Controls (Mean)	Patients (Mean)	*p*-Value	Controls (Mean)	Patients (Mean)	*p*-Value
Basal	42.7	36.3	0.004	44.5	29.7	<0.0001	49.6	37.3	0.0006
MR	118.9	93.6	<0.0001	102.6	76.7		136	102.7	0.002
SRC	78.6	59.9	0.0003	68.8	46.8	0.006	92.3	56.9	<0.0001
ATP	34.2	33.2		30.9	22.6	0.009	40.2	33.7	0.04

**Table 3:** The effect of treatment of patients (*n* = 5) and controls (*n* = 5) myoblasts with 300 µM palmitate or oleate conjugated with BSA on key parameters for mitochondrial function (basal, MR, ATP production and SRC). Significant differences between values obtained with or without FAs are presented if *p* ≤ 0.05. *p*.: *p*-values, –FAs: without Fatty acids, Pa: palmitate. O: oleate.

**Controls**								
	**–FAs** **(Mean)**	**Pa** **(Mean)**	***p.* –FAs/Pa** **(Mean)**	**O** **(Mean)**	***p.* –FAs/O** **(Mean)**	**Pa** **(Mean)**	**O** **(Mean)**	***p.* Pa/O** **(Mean)**
Basal	42.7	44.5		49.6		44.5	49.6	
MR	118.9	102.6		136		102.6	136	0.01
SRC	78.6	68.9		92.3		68.9	92.3	0.01
ATP	34.2	30.9		40.2	0.02	30.9	40.2	0.003
**Patients**								
	**–FAs** **(Mean)**	**Pa** **(Mean)**	***p.* –FAs/Pa** **(Mean)**	**O** **(Mean)**	***p.* –FAs/O** **(Mean)**	**Pa** **(Mean)**	**O** **(Mean)**	***p.* Pa/O** **(Mean)**
Basal	36.3	29.7	0.03	37.3		29.7	37.3	0.04
MR	93.6	76.7		102.7		76.7	102.7	0.02
SRC	59.9	46.8	0.04	56.9		46.8	56.9	
ATP	33.2	22.6	<0.0001	33.7		22.6	33.7	<0.0001

**Table 4:** Measurement of ß-oxidation using a Seahorse XF96 Cell Analyzer in myoblasts of patients (*n* = 5) and controls (*n* = 5) with palmitate or oleate as substrate at 37 °C and 40 °C. Significance was set at *p* = 0.05. FAO: fatty acid oxidation.

**FAO 37 °C**						
	**Palmitate as Substrate**			**Oleate as Substrate**		
	**Controls (Mean)**	**Patients (Mean)**	***p*-Value**	**Controls (Mean)**	**Patients (Mean)**	***p*-Value**
Basal	28.4	23.7	0.04	26.3	23.5	
MR	70.7	64.9		85.9	73.7	
SRC	46	45.7		62.8	55.5	
ATP	21.1	20.2		21.1	20.6	
**FAO 40 °C**						
	**Palmitate as Substrate**			**Oleate as Substrate**		
	**Controls (Mean)**	**Patients (Mean)**	***p*-Value**	**Controls (Mean)**	**Patients (Mean)**	***p*-Value**
Basal	17.2	17.5		18.8	17.9	
MR	45.1	38		49.5	44.3	
SRC	28	20.8		35.5	33.3	
ATP	12	11.7		13.9	13.9	

**Table 5:** Evaluation of ß-oxidation using a Seahorse XF96 Cell Analyzer in myoblasts of patients (*n* = 5) and controls (*n* = 5) with palmitate or oleate as substrate. The difference between values obtained at 37 °C and 40 °C is shown if the results were significant (*p*-values ≤0.05).

**Controls**						
	**Palmitate as Substrate**			**Oleate as Substrate**		
	**37 °C (Mean)**	**40 °C (Mean)**	***p*-Value**	**37 °C (Mean)**	**40 °C (Mean)**	***p*-Value**
Basal	28.4	17.2	<0.0001	26.3	18.8	0.005
MR	70.7	45.1	0.006	85.9	49.5	<0.0001
SRC	46	28	0.05	62.8	35.5	0.0002
ATP	21.1	12	<0.0001	21.1	13.9	0.001
**Patients**						
	**Palmitate as Substrate**			**Oleate as Substrate**		
	**37 °C (Mean)**	**40 °C (Mean)**	***p*-Value**	**37 °C (Mean)**	**40 °C (Mean)**	***p*-Value**
Basal	23.7	17.5	0.02	23.5	17.9	0.04
MR	64.9	38	0.0006	73.7	44.3	0.0003
SRC	45.7	20.8	0.0006	55.5	33.3	0.003
ATP	20.2	11.7	<0.0001	20.6	13.9	0.004

**Table 6:** Evaluation of ß-oxidation using a Seahorse XF96 Cell Analyzer at 37 °C and 40 °C in myoblasts of patients (*n* = 5) and controls (*n* = 5) with palmitate or oleate as substrate. The difference between values obtained using palmitate or oleate as substrate is shown if the results were significant (*p*-values ≤ 0.05), Pa: palmitate. O: oleate.

**FAO 37 °C**						
	**Controls**			**Patients**		
	**Pa (Mean)**	**O (Mean)**	***p*-Value**	**Pa (Mean)**	**O (Mean)**	***p*-Value**
Basal	28.4	26.3		23.7	23.5	
MR	70.7	85.9		64.9	73.7	
SRC	46	62.8	0.04	45.7	55.5	
ATP	21.1	21.1		20.2	20.6	
**FAO 40 °C**						
	**Controls**			**Patients**		
	**Pa (Mean)**	**O (Mean)**	***p*-Value**	**Pa (Mean)**	**O (Mean)**	***p*-Value**
Basal	17.2	18.8		17.5	17.9	
MR	45.1	49.5		38	44.3	
SRC	28	35.5		20.8	33.3	
ATP	12	13.9		11.7	13.9	
